# Sex Hormone Binding Globulin (SHBG) Mitigates ER Stress in Hepatocytes In Vitro and Ex Vivo

**DOI:** 10.3390/cells10040755

**Published:** 2021-03-30

**Authors:** Katarzyna Kornicka-Garbowska, Lynda Bourebaba, Michael Röcken, Krzysztof Marycz

**Affiliations:** 1 Department of Experimental Biology, Faculty of Biology and Animal Science, Wrocław University of Environmental and Life Sciences, Norwida 27B Street, A7 Building, 50-375 Wrocław, Poland; kornicka.katarzyna@gmail.com (K.K.-G.); lynda.bourebaba@upwr.edu.pl (L.B.); 2 International Institute of Translational Medicine, Jesionowa, 11, Malin, 55-114 Wisznia Mała, Poland; 3 Faculty of Veterinary Medicine, Equine Clinic—Equine Surgery, Justus-Liebig-University, 35392 Gießen, Germany; Michael.Roecken@vetmed.uni-giessen.de

**Keywords:** sex hormone binding globulin, SHBG, liver, metabolic syndrome, endoplasmic reticulum stress

## Abstract

Despite multiple research studies regarding metabolic syndrome and diabetes, the full picture of their molecular background and pathogenies remains elusive. The latest studies revealed that sex hormone-binding globulin (SHBG)—a serum protein released mainly by the liver—may participate in metabolic dysregulation, as its low serum level correlates with a risk for obesity, metabolic syndrome, and diabetes. Yet, the molecular phenomenon linking SHBG with these disorders remains unclear. In the presented study, we investigate how exogenous SHBG affects metabolically impaired hepatocytes with special attention to endoplasmic reticulum stress (ER stress) and lipid metabolism both in vitro and ex vivo. For that reason, palmitate-treated HepG2 cells and liver tissue samples collected post mortem were cultured in the presence of 50 nM and 100 nM SHBG. We found that SHBG protects against ER stress development and its progression. We have found that SHBG decreased the expression levels of inositol-requiring enzyme 1 (IRE1α), activating transcription factor 6 (ATF6), DNA damage-inducible transcript 3 (CHOP), and immunoglobulin heavy chain-binding protein (BIP). Furthermore, we have shown that it regulates lipolytic gene expression ex vivo. Additionally, herein, we deliver a novel large-animal model to study SHBG in translational research. Our data provide new insights into the cellular and molecular mechanisms by which SHBG modulates hepatocyte metabolism and offer a new experimental approach to study SHBG in human diseases.

## 1. Introduction

Metabolic syndrome in human beings (MetS) and equine metabolic syndrome (MS) are increasingly frequently diagnosed endocrine disorders that develop due to sedentary lifestyle, lack of physical activity, and overfeeding with nonstructural carbohydrates [1]. As estimated by National Health and Nutrition Examination Survey (NHANES), more than one-third of all US adults fulfill the criteria and definition for MetS as agreed by several organizations [2]. The same situation has been reported for horses, only in the United Kingdom (UK), 30% of the horse population exhibit symptoms of MS [3,4]. Pathogenesis and clinical symptoms of both conditions are closely related, which supports the application of equid as a large animal model to study human metabolic disorders [1,5]. This equine model represents a unique and valuable system for translational research. MetS and MS are characterized by a cluster of biological and clinical factors that include obesity (adiposity–MS), insulin resistance (IR), lipotoxicicty, and inflammation [6,7]. The progression of both MetS and MS may lead to the development of related complications, which include cardiovascular diseases (in MS laminitis) [8].

Recently, increasing attention has been paid to understanding the role of liver deterioration during IR, metabolic dysfunction, and lipotoxicicty, as these represent a crucial component of MetS and MS [9,10]. Progressive IR in peripheral tissues leads to increased accumulation of free fatty acids (FFA) in the liver, and in turn enhances triglyceride and very low-density lipoprotein (VLDL) synthesis in hepatocytes. The accumulation of FFA and in particular overload with saturated fatty acids (SFA) trigger lipotoxicicty, lipoapoptosis, and finally IR development in the liver [11,12]. As a consequence, fatty degeneration in hepatocytes and increased levels of C-reactive protein (CRP) in circulating blood initiate the development of endoplasmic reticulum stress (ER stress) [13].

The endoplasmic reticulum (ER) is a cellular organelle that plays a pivotal role in hepatocytes due to the high metabolic rates of these cells, which include protein synthesis and processing, lipid synthesis, and calcium storage. The excessive accumulation of defective proteins (misfolded or unfolded) in the ER lumen or calcium depletion may lead to ER stress, apoptosis, as well as inflammation [14]. Moreover, it has been shown that in response to ER stress, caspase-2 becomes activated and initiates lipoapoptosis, which is a cell death pathway triggered by excessive intracellular accumulation of lipids. In response to ER stress, an adaptive cellular response termed the unfolded protein response (UPR) aims to restore ER homeostasis and promote cell survival. UPR signaling is mediated via three distinct pathways [15]. These rely on one of the three sensors including inositol-requiring enzyme 1 (IRE1α), protein kinase RNA-like endoplasmic reticulum kinase (PERK), and activating transcription factor 6 (ATF6). The activation of ATF6 in response to increased intramembrane proteolysis (RIP) is mediated by its cleavage, which in turn produces an N-terminal cleaved product of ATF6 (p50ATF6). As a consequence, the spliced forms of X-box binding protein 1 (sXBP1) and p50ATF6 trigger the synthesis of ER chaperones such as immunoglobulin heavy chain-binding protein (BIP, also referred as GRP78), which reduces the accumulation of unfolded protein by assisting with protein folding [16]. However, this mechanism fails when exposure to ER stress is prolonged and cellular death mechanisms are already triggered. The deterioration of hepatocytes by the initiation of ER stress is closely related to the development of IR and accompanies MetS and MS. Therefore, targeting ER stress may become a valuable strategy against metabolic disorders development.

Recently, sex hormone-binding globulin (SHBG), a glycoprotein with a molecular mass about 90 kDa that is primarily synthesized in the liver, has been implicated in the development of metabolic disorders [17]. Several studies have indicated that beyond the regulation of circulating hormone concentrations and their transport to target tissues, SHBG exerts additional yet not fully understood functions [18,19,20,21]. It was shown that free SHBG binds to its receptor (SHBG-R) or directly to the plasma membrane of different cells and different tissues, i.e., endometrium, prostate, liver, and kidney [22,23]. Additionally, the insulin-resistant state is associated with low SHBG expression, while higher levels of SHBG are associated with lower risk of diabetes development [24]. It was demonstrated that SHBG is negatively correlated with IR and becomes an independent risk factor for IR in women with gestational diabetes mellitus [25,26].

The results of previous studies led to us to investigate whether the compensation of SHBG is able to mitigate the ER stress axis in insulin-resistant hepatocytes and liver sections. We decided to perform the experiments using the HepG2 cell line as its well establish a model to study insulin resistance, and what is more, these cells synthetize and secrete SHBG [27,28]. Here, we have found that SHBG in vitro and ex vivo reduces hepatocyte ER stress by the amelioration of lipogenesis. Herein, we provide the evidence that decreased SHBG levels in the liver of insulin-resistant metabolic syndrome individuals are related to ER stress and that the application of exogenous SHBG prior to the compensation of its physiological level can protect against that harmful mechanism.

## 2. Materials and Methods

All reagents were purchased from Sigma Aldrich (Munich, Germany) unless indicated otherwise.

### 2.1. Cell Culture

The HepG2 cell line was purchased from ATCC (HB-8065) and cultured in accordance to the manufacturer’s protocol. Cell culture medium consisted of Dulbecco’s Modified Eagle’s Medium (DMEM), low glucose w/l-glutamine, w/sodium pyruvate (Biowest, Riverside, MO, USA) supplemented with 10% Fetal Bovine Serum (FBS, Sigma Aldrich, Munich, Germany). Cells were cultured in aseptic and unchanging conditions in an incubator (37 °C in a humidified 5% CO_2_ atmosphere). Cells were passaged with TrypLE Express solution (ThermoFisher, Warsaw, Poland). The media were changed every second day.

### 2.2. Experimental Protocol

Prior to the induction of IR with palmitate, cells were first pretreated with native SHBG (Abcam, Cambridge, UK, ab151275) at a concentration of 50 and 100 nM for 24 h. Protein was diluted in DMEM containing 0.2% fatty acid-free bovine serum albumin (Serva, Heidelberg, Germany). In accordance to the manufacturer, native SHBG contamination with sex hormones was at a very low level. It was confirmed by the recent research which showed that the studied SHBG solution contains negligible amounts of sex steroids (Abcam’s SHBG protein contained molar ratios of 1:5600 and 1:10,000 for testosterone and estradiol, respectively, to SHBG) so it is unlikely that these hormones contribute to the results obtained with that product. Cells treated with BSA alone (0.2%) served as control.

To induce IR and lipotoxicicty in cells, they were treated with the combination of palmitate albumin in accordance to the protocol established by Yang et al. [29]. Briefly, sodium palmitate (PA, C16:0) was dissolved in 0.1 M sodium hydroxide by heating at 70 °C and then diluted with 10% fatty acid-free BSA to the concentration of 5 mM. Then, the diluted solution was incubated for 10 min at 55 °C. The obtained solution (PA-BSA complex) was finally filtered with a 0.22 µm syringe filter prior to decontamination. The complex was diluted in DMEM at a final concentration of 0.6 mM and added to cells. Cells in the control group were treated with a respective amount of BSA only. Before adding palmitate to experimental group, medium with SHBG was removed.

### 2.3. Staining of Intracellular Lipid

To visualize the accumulation of lipid droplets within the cells after PA treatment, they were stained with Oil Red O dye. Prior to staining, cells were washed with phosphate buffer saline (PBS) and fixed with 4% paraformaldehyde (PFA). An additional wash was performed to remove the fixation solution, and cells were treated with 60% isopropanol for 5 min. After the removal of alcohol and washing, cells were incubated with Oil Red O solution for 15 min at room temperature (RT). Specimens were observed under an inverted microscope (Leica), and pictures were acquired using a Canon PowerShot digital camera. Accumulation of dye was quantified using spectrophotometrical measurement. Briefly, the accumulated dye was washed out from the cells using 100% isopropanol and subjected to 96-well plates for the measurement of absorbance at 490 nm/570 nm wavelengths using a plate reader (Epoch, Biotek, Bad Friedrichshall, Germany).

Alternatively, neutral lipid accumulation was observed in cells using Lipid Tox™ Green Neutral Lipid Stain (ThermoFisher). Fixed samples were incubated with the dye solution (1:200) for 30 min at RT. After washing, the dye, the specimens were mounted on glass slides using ProLong™ Diamond Antifade Mountant with DAPI (ThermoFisher) and observed in a confocal microscope (Leica TCS SPE).

### 2.4. Visualization of ER

Prior to staining, cells were seeded onto glass coverslips at a density of 5.0 × 10^5^ cells/cm^2^. Then, in accordance with the experimental protocol, cells were treated with SHBG and palmitate for 6 and 24 h, respectively. Then, the medium was removed and replaced with a 1 µM solution of ER-TrackerTM Green (ThermoFisher) diluted in PBS. Cells were treated with dye for 30 min at 37 °C, 5% CO_2_. Subsequently, cells were fixed in 4% PFA and mounted on glass slides using ProLong™ Diamond Antifade Mountant with DAPI (ThermoFisher). Samples were observed under confocal microscope (Leica TCS SPE).

### 2.5. Immunofluorescence

Prior to immunofluorescent staining, cells were seeded on glass coverslips at a density of 5.0 × 10^5^ cells/cm^2^. Then, the samples underwent an experimental protocol based on SHBG and PA treatment as described above. Prior to staining, cells were (i) fixed with 4% PFA for 45 min at RT, (ii) treated with 0.2% Tween 20 in PBS for 15 min, (iii) blocked with 10% goat serum, (iv) incubated with the respective primary antibodies diluted at 1:100 overnight at 4 °C with XBP1-x-box binding protein 1, Aviva System Biology, (v) incubated with Atto 488-labeled secondary antibodies diluted at 1:1000, and (vi) mounted onto glass slides with ProLong™ Diamond Antifade Mountant with DAPI (ThermoFisher, Warsaw, Poland ). Images were acquired with a confocal microscope (Leica TCS SPE, KAWA.SKA Sp. z o.o., Zalesie Gorne, Poland) and analyzed with ImageJ software (Wayne Rasband, National Institute of Health, USA).

### 2.6. Immunoblotting

Cells were homogenized in radioimmunoprecipitation assay buffer (RIPA) supplemented with Protease Phosphatase Inhibitor Cocktail on ice. Next, samples were sonicated for 10 s and centrifuged (12,000× *g* for 20 min). Protein amount was established with Pierce™ BCA Protein Assay Kit (ThermoFisher). Samples were solubilized with Laemmli buffer (Bio-Rad) and equaled amounts of proteins from whole cell lysates were subjected to SDS-PAGE. Next, proteins were transferred to a polyvinylidene difluoride membranę (PVDF, Merck Milipore, Darmstadt, Germany), blocked with Tris-buffered saline with 0.1% Tween ® 20 Detergent (TBST) buffer with 5% bovine serum albumin (BSA) solution for 1 h at room temperature (RT), reacted with primary antibodies, and subsequently with horse radish peroxidase (HRP)-labeled secondary antibodies. Samples were probed with SuperSignal™ West Pico PLUS Chemiluminescent Substrate (ThermoFisher), imaged using ChemiDoc MP Imaging System (Bio-Rad, Hercules, CA, USA), and quantified with Image Lab Software (Bio-Rad). Antibodies applied in Western blotting are listed in Table 1.

### 2.7. Quantitative Reverse-Transcription Polymerase Chain Reaction (qRT-PCR)

Total RNA was isolated from cells using Extrazol^®^ (Blirt DNA) in accordance with the method established by Chomczynski and Sacchi [30]. Reverse transcription was performed with Tetro cDNA Synthesis Kit (Bioline Reagents Ltd., London, UK) using T100 Thermal Cycler (Bio-Rad). qRT-PCR analysis was performed with SensiFAST SYBR^®^&Fluorescein Kit (Bioline Reagents Ltd., London, UK) using CFX Connect Real-Time PCR Detection System (Bio-Rad). The GAPDH levels served as an internal control for the normalization of the expression of all investigated genes. Gene expression was quantified using the 2^−ΔΔCT^ method. Xbp1 splicing was determined by running the qRT-PCR product in 3% agarose gel. Sequences of primers used in qRT-PCR are listed in Table 2.

Prior to miRNA analysis, total RNA was polyadenylated and converted to cDNA with a Mir-X miRNA First-Strand Synthesis Kit (Takara) in accordance with the manufacturer’s instructions. The expression data were normalized using the 2^−ΔΔCT^ method in relation to the U6 snRNA used as a housekeeping gene. Primer sequences are summarized in Table 3.

### 2.8. Animal Qualification and Preparation of Liver Ex Vivo Specimens

Eight MS and eight healthy horses were selected from a slaughterhouse located in Rawicz, Poland, and samples of liver were collected post mortem as described previously [11]. The horses, which were qualified for that research, were Polish warmblood horses of both sexes, aged between 9 and 14 years old. MS was diagnosed on the basis of the criteria established in the 2010 American College of Veterinary. MS horses were characterized on the basis of insulin dysregulation (ID), body condition scoring (BCS), regional adiposity, weight (Wt), and history of laminitis. The detailed characterization of animals can be found in our previous paper [11]. Liver samples of animals were subjected to a transmission electron microscope following the protocol published previously [11]. Liver samples were transferred to Hank’s solution with 5 mM glucose. Then, small tissue samples (5 mm) collected from animals post mortem were obtained with 21 gauge needles. Then, specimens were cut manually to 1 mm and cultured in Dulbecco’s Modified Eagle’s Medium (DMEM), low glucose w/l-glutamine, w/sodium pyruvate (Biowest, Riverside, MO, USA) supplemented with 10% FBS (Sigma Aldrich, Munich, Germany). Cells were cultured in aseptic and unchanging conditions in an incubator (37 °C in a humidified 5% CO_2_ atmosphere). Samples harvested from MS were additionally assigned to experimental groups in which they were treated with native SHBG (Abcam, ab151275) at a concentration of 50 and 100 nM for 6 and 24 h. Then, the samples were subjected to further analysis.

### 2.9. ELISA Assays

The total concentration of proteins in the serum of animals was estimated with enzyme-linked immunosorbent assay (ELISA) for interleukin 6 (IL-6) (My Biosource, San Diego, CA, USA) and peroxisome proliferator-activated receptor gamma (PPARG) (My Biosource, San Diego, CA, USA). Assays were performed in accordance with the manufacturer’s protocol. Spectrophotometric determination was performed with Epoch BioTek^®^ (Bad Friedrichshall, Germany).

### 2.10. Statistics

All data are presented as the means  ±  standard deviation (S.D.). The differences between groups were analyzed with the one-way analysis test (ANOVA and nonparametric) followed by Tukey’s post hoc test in GraphPad Software (Prism 8.20). *p*  <  0.05 was considered to indicate a statistically significant difference.

## 3. Results

### 3.1. The Effect of Lipid Overload on SHBG Levels

**Figure 1:**
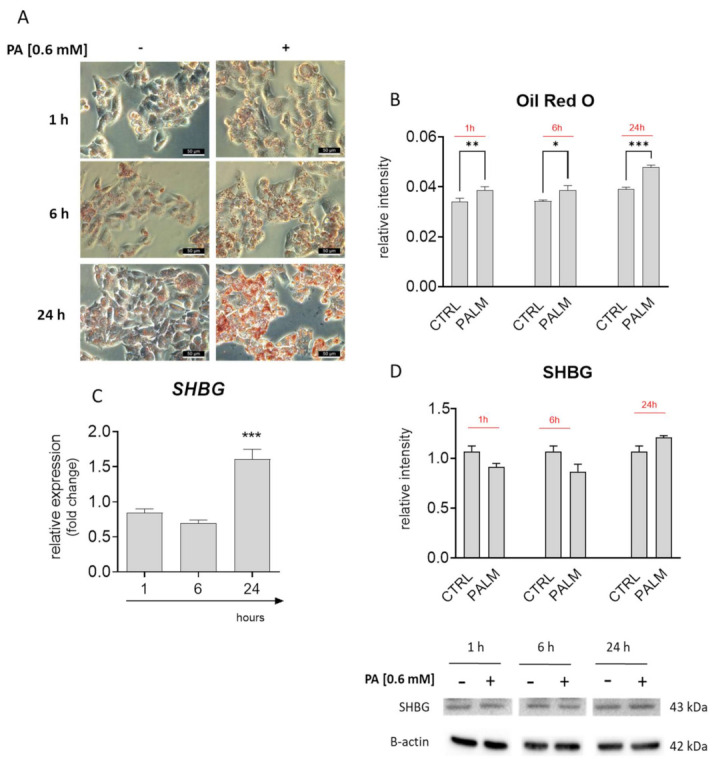
Correlation between lipid overload and sex hormone-binding globulin (SHBG) levels in palmitate (PA) treated HepG2 cells. Oil Red O staining (**A**) and its quantification (**B**) revealed time-depended lipid overload. PA treatment significantly upregulated the SHBG expression (**C**); however, the protein amount remained unchanged (**D**). The results are presented as mean ± SEM; *n* = 6, * *p* < 0.05, ** *p* < 0.01, *** *p* < 0.001.

In the presented study, to induce lipotoxicicty and IR, cells were treated with palmitate (PA), which is a well-established cellular model to study metabolic dysregulation in cells. Prior to the experiments, cells were treated with PA at a concentration of 0.6, mM for 1, 6, and 24 h. To confirm lipid overload, cells were stained with Oil Red O (Figure 1A). Furthermore, stainings were quantified (Figure 1B), revealing that the greatest accumulation of PA occurs 24 h post treatment. Next, using RT-qPCR, a substantial decrease of SHBG expression was shown after 6 h of PA treatment (Figure 1C). Interestingly, the mRNA levels of SHBG were significantly upregulated after 24 h; however, the protein levels remained unchanged (without statistical significance) (Figure 1C). These prompt us to treat cells with PA in the next experiments for 24 h. We hypothesize that due to lipid overload, cells trigger an enhanced expression of SHBG as a rescue mechanism; however, as a consequence of disturbances in cellular metabolism, protein synthesis is blocked. Thus, in the next experiments, we decided to deliver exogenous SHBG to HepG2 to investigate whether compensation of its levels protects cells against PA-induced lipotoxicicty and IR.

### 3.2. The Effects of SHBG on Lipid Metabolism

**Figure 2:**
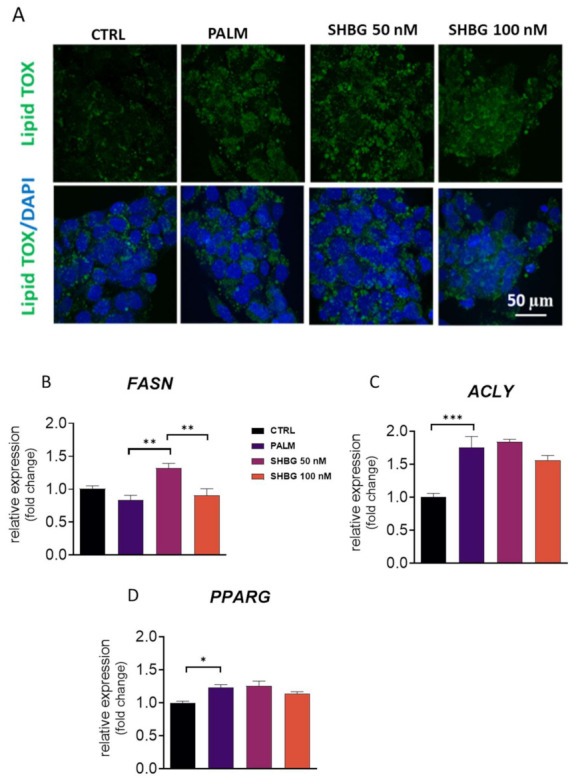
The effects of SHBG on lipid metabolism. Cells were pretreated with SHBG at concentrations of 50 and 100 nM in serum-free DMEM medium for 24 h before exposition to PA for 24 h. Accumulation of neutral lipids was visualized with HCS LipidTOX™ Green staining and confocal microscope (**A**). Transcript levels of lipogenic enzymes FASN (**B**), ACLY (**C**), and PPARG (**D**) were established by RT-qPCR. The results are presented as mean ± SEM; *n* = 6, * *p* < 0.05, ** *p* < 0.01, *** *p* < 0.001.

Prior to RT-qPCR analysis, cell cells were pretreated with SHBG at concentrations of 50 and 100 nM for 24 h followed by stimulation with PA for 24 h. Staining with HCS LipidTOX™ Green revealed that PA markedly enhanced the accumulation of lipids in a time-dependent manner (Figure 2A); however, it is not clear from photographs that pretreatment of cells with SHBG protects against lipid overload. For that reason, we investigated the expression of enzymes involved in de novo lipogenesis: FASN and ACLY. Obtained results revealed that SHBG pretreatment enhanced FASN (Figure 2B) expression, while ACLY (Figure 2C) mRNA levels remained unchanged. Furthermore, we observed an upregulation of PPARG (Figure 2D) after PA treatment, and SHBG pretreatment did not influence its levels. The obtained data indicate that SHBG exerts only limited action on lipid metabolism in HepG2 cells treated with PA.

### 3.3. SHBG Protects against PA-Induced ER Stress

**Figure 3:**
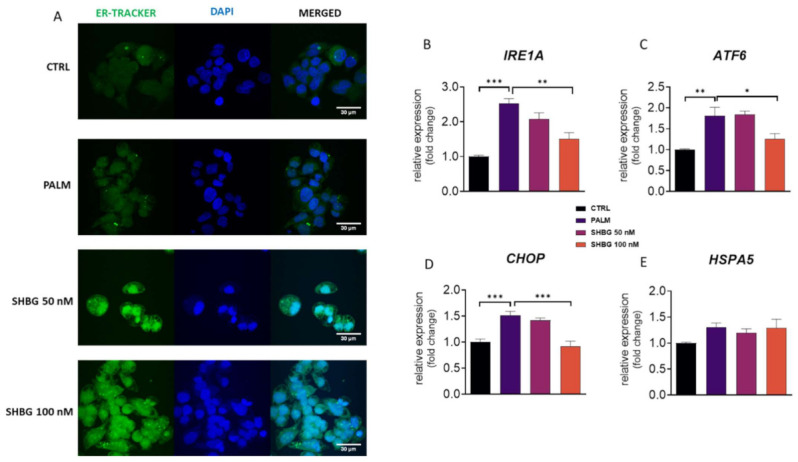

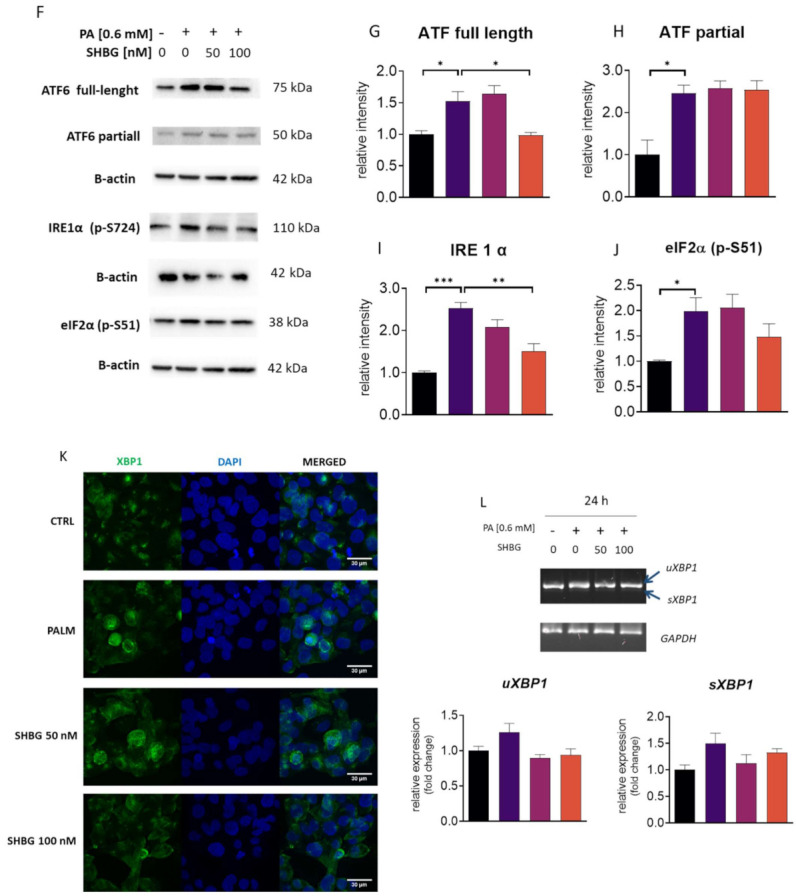
SHBG protects against PA-induced endoplasmic reticulum (ER) stress. HepG2 cells were incubated with SHBG in two concentrations (50 or 100 nM) before exposition to PA for 24 h. ER localization in cells was visualized with ER-TrackerTM Green (**A**). mRNA levels of IRE1A (**B**), ATF6 (**C**), CHOP (**D**), and HSPA5 (**E**) were determined using the RT-qPCR method. The phosphorylation of eIF2α at Ser51 and IRE1α at Ser724, as well as the cleavage activation of ATF6 were estimated using the Western blot technique (**F**). Relative intensities of ATF6 full length (**G**), ATF6 cleaved (**H**), phosphorylated IRE1α (**I**), and eIF2α (**J**) were calculated with Image Lab software after normalization to β-actin as a control housekeeping protein. Intracellular localization of XBP1 investigated with confocal microscopy (**K**). Expression levels (**L**) of spliced and unsliced XBP1 established by RT-qPCR followed by gel electrophoresis. The results are presented as mean ± SEM; *n* = 6, * *p* < 0.05, ** *p* < 0.01, *** *p* < 0.001.

ER stress and its consequences trigger in cells a UPR response, which is involved in the pathogenesis of IR and non-alcoholic fatty liver disease (NAFLD). To evaluate whether SHBG may protect against the aggravation of PA-induced ER stress, HEpG2 cells were pretreated with SHBG in concentrations of 50 and 100 nM in serum-free DMEM for 24 h before stimulation with PA (0.6 mM) 24 h. To visualize ER in the investigated cells, they were stained with ER-TrackerTM Green (Figure 3A). Pre-treatment with SHBG resulted in an enhanced fluorescent signal originating from the ER net, which may suggest that its functionality remains or indicate its high metabolic activity. For that reason, in the next experiments, we decided to evaluate the expression of key UPR-linked markers. We have found that SHBG pretreatment significantly decreased the mRNA levels of IRE1A (Figure 3B), ATF6 (Figure 3C), and CHOP (Figure 3D) after PA application. No significant changes in the expression of HSPA5 (Figure 3E) were noticed. To confirm the protective role of SHBG on PA-induced ER stress, we decided to evaluate the phosphorylation of key UPR markers, such as IRE1α, eIF2α, and ATF6 that indicate UPR activation. Representative bands from Western blots are shown in Figure 3F. Data quantification revealed that PA treatment increased the amount of full-length (Figure 3G) and partial ATF (Figure 3H); however, pre-treatment of cells with SHBG resulted in decreased levels of full-length form. Similarly, enhanced phosphorylation of eIF2α was found in PA-treated cells (Figure 3I); however, SHBG at a concentration of 100 nM significantly reduced its phosphorylation. We observed enhanced phosphorylation of eIF2α in the PA group (Figure 3J), and no changes in its levels were found after SHBG application. Furthermore, protein expression and cellular distribution of XBP1 using immunofluorescence were determined. We observed a significant increase in XBP1 level in HepG2 upon PA treatment (Figure 3K) and its reduced levels in cells pretreated with 100 nM SHBG. Protein was predominantly localized in the nucleus after 24 h post-PA treatment, while the nuclear localization of XBP1 was reduced in HepG2 after pretreatment with SHBG at the concentration of 100 nM SHBG. Additionally, to determine the changes in XBP1 alternatives splicing, PCR products were run on 3% agarose gel and quantified (Figure 3L). No significant changes of alternative XBP1 splicing were found due to SHBG pretreatment.

### 3.4. SHBG Pretreatment Affects miRNA Profile

**Figure 4:**
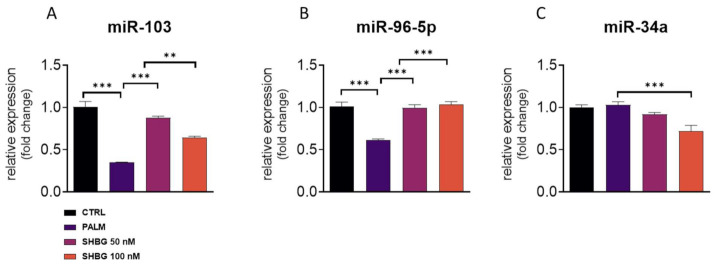
SHBG pretreatment affects miRNA profile. HepG2 cells were incubated with SHBG at two concentrations (50 and 100 nM) for 24 h followed by stimulation with PA (0.6 mM) for 24 h. The levels of miR-103 (**A**), miR-96-5p (**B**), and miR-34a (**C**) were determined using RT-qPCR method and U6 snRNA was used as a housekeeping gene. The results are presented as mean ± SEM; ** *p* < 0.01, *** *p* < 0.001.

The activation of UPR and particularly, IRE1α activation leads to the rapid decay of selected microRNAs. For that reason, we established the expression of miRNAs related to ER stress and apoptosis in treated cells. We have found that PA treatment significantly downregulated the amount of miR-103 (Figure 4A) and miR-96-5p (Figure 4B), although SHBG pretreatment reversed that phenomenon. Interestingly, the difference in miR-34a levels was only found in cells treated with 100 nM SHBG (Figure 4C).

### 3.5. Liver of MS Individuals Suffers from Lipotoxicicty, ER Stress, and Decreased SHBG Levels

**Figure 5:**
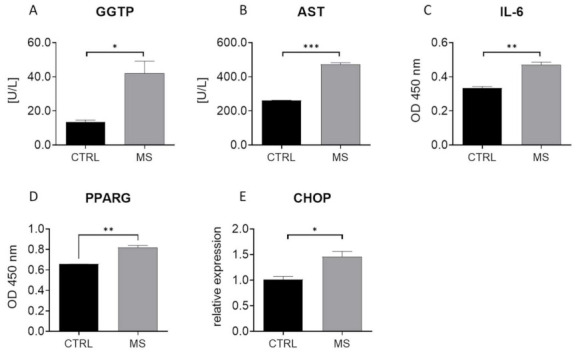

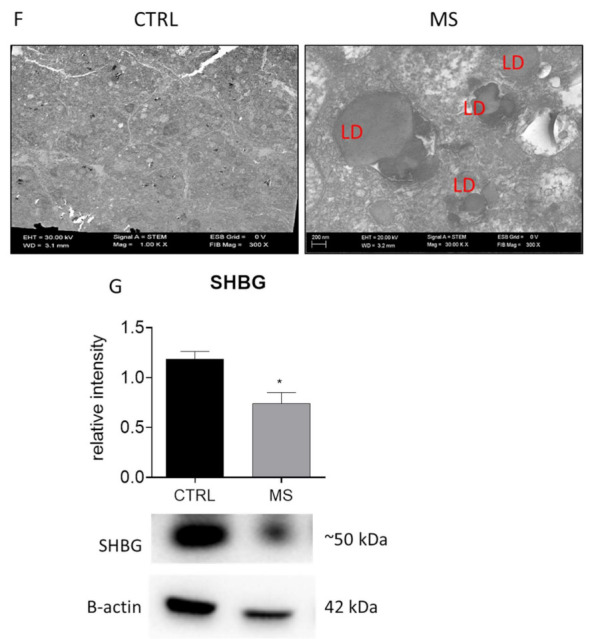
MS individuals are characterized by decreased liver function and suffer from lipotoxicicty, ER stress, and decreased SHBG levels. Biochemical analysis revealed increased levels of GGTP (**A**) and AST (**B**) in the circulating blood of MS subjects. Furthermore, using ELISA, the amount of IL-6 (**C**) and PPARG (**D**) in serum was evaluated. RT-qPCR revealed an increased expression of CHOP (**E**) and ultrastructural alternations including lipid overload (**F**) in metabolic syndrome-derived liver samples. These specimens were also characterized by decreased SHBG levels (**G**). The results are presented as mean ± SEM; *n* = 6, * *p* < 0.05, ** *p* < 0.01, *** *p* < 0.001.

To establish the model for the ex vivo part of the research, we investigated the metabolic characteristics of liver from MS individuals. Obtained results revealed increased levels of GGTP (Figure 5A) and AST (Figure 5B) as well as proinflammatory IL-6 (Figure 5C) and PPARG (Figure 5D) in their circulating blood. The increased expression of CHOP (Figure 5E) and increased accumulation of lipid droplets (Figure 5F) were found in liver tissue from MS individuals. Furthermore, decreased liver synthesis of SHBG was observed (Figure 5G).

### 3.6. The Effects of SHBG on Lipolytic Gene Expression Ex Vivo

**Figure 6:**
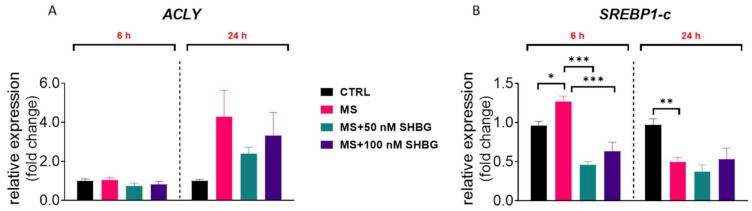
The effects of SHBG on lipolytic gene expression ex vivo. To evaluate how exogenous SHBG affects the expression of lipid metabolism-related genes in hepatocytes, liver samples collected from animals *post mortem* (healthy: CTRL and metabolic syndrome individuals: MS) were maintained ex vivo in the presence of 50 and 100 nM SHBG for 6 and 24 h. mRNA levels of ACLY (**A**) and SREBP1-C (**B**) were determined using the RT-qPCR method. The results are presented as mean ± SEM; *n* = 6, * *p* < 0.05, ** *p* < 0.01, *** *p* < 0.001.

To evaluate how exogenous SHBG affects the expression of lipid metabolism-related genes in hepatocytes, liver fragments collected from tissue samples were cultured ex vivo in the presence of 50 and 100 nM SHBG for 6 and 24 h. ACLY upregulation is found in the liver of obese, fatty liver, and type two diabetes individuals; however, in the presented study, we did not observe any statistically significant differences in its mRNA levels between the investigated groups (Figure 6A). However, a slight decrease in its expression was observed after 24 h of culture in sections treated with 50 nM SHBG in comparison to untreated counterparts. Interestingly, we observed upregulation after 6 h of culture, while downregulation after 24 h of the expression of SREBP1-c, which downregulates hepatic gluconeogenesis and has been reported to be upregulated in obese animals (Figure 6B). Interestingly, treatment with SHBG resulted in downregulation of its expression after 6 h.

### 3.7. SHBG Reduces ER Stress in Liver Sections Ex Vivo

**Figure 7:**
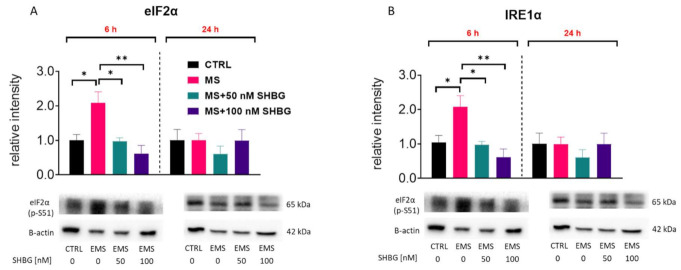

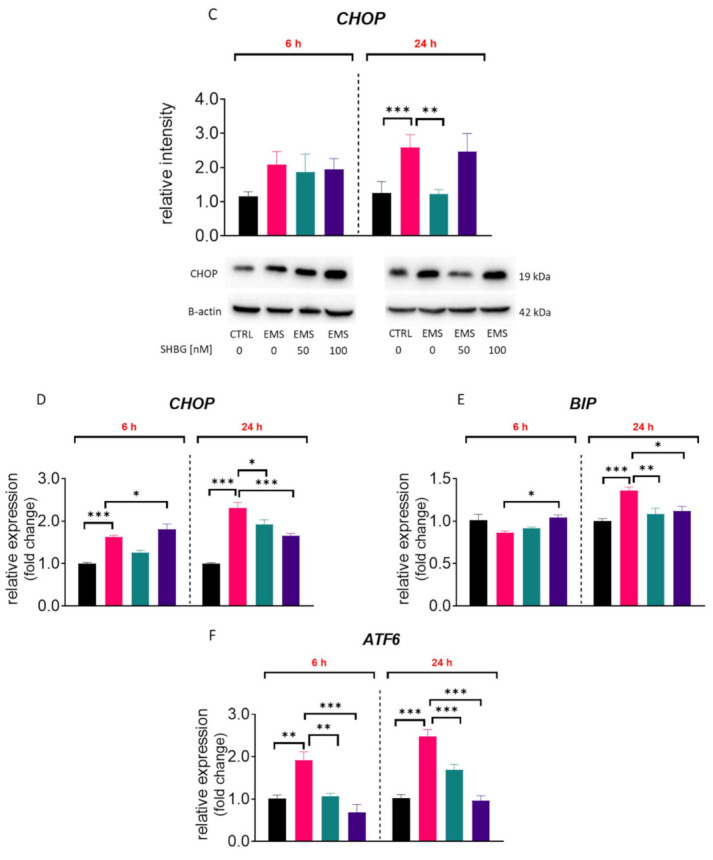

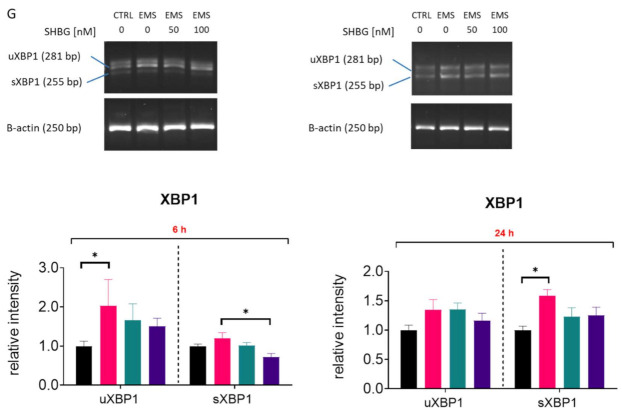
SHBG reduces ER stress in liver sections ex vivo. To confirm the protective effects of SHBG on liver sections we treated them with 50 and 100 nM SHBG for 6 and 24 h respectively. The phosphorylation of key UPR markers, such as IRE1α (**A**) and eIF2α (**B**) as well as CHOP (**C**) levels were determined by Western blot. Furthermore, the expression of CHOP (**D**), BIP (**E**), and ATF6 (**F**) was established by RT-qPCR. mRNA levels of unspliced (uXBP1) and spliced (sXBP1) XBP1 were evaluated by RT-PCR products were run on the 3% agarose gel. The relative amount of both XBP1 forms was determined using Image Lab software after normalization with GAPDH as a reference gene (**G**). The results are presented as mean ± SEM; *n* = 6, * *p* < 0.05, ** *p* < 0.01, *** *p* < 0.001.

To confirm the protective effects of SHBG against ER stress not only in vitro but also ex vivo, liver fragments acquired from tissue samples were cultured in the presence of 50 and 100 nM SHBG for 6 and 24 h. The phosphorylation of key UPR markers, such as IRE1α and eIF2α, as well as CHOP levels were determined by Western blot. Obtained results revealed that the phosphorylation of both eIF2α at Ser51 (Figure 7A) and IRE1α at Ser724 (Figure 7B) was enhanced in MS livers. Yet, the application of SHBG for 6 h reduces the phosphorylation of these proteins. No statistically significant differences were in CHOP levels between the investigated groups (Figure 7C). Slightly reduced levels of that protein (but without statistical significance) were observed in MS livers treated with 50 nM SHBG for 24 h. For that reason, we also tested the mRNA levels of CHOP with RT-qPCR (Figure 7D). Obtained results revealed its enhanced expression in MS samples, which was reduced after 24 h of culture with SHBG. The expression of BIP was downregulated after 24 h of culture with SHBG (Figure 7E), while ATF6 mRNA levels (Figure 7F) were decreased in both time points. To determine the changes in XBP1 alternative splicing, the PCR products were run on 3% agarose gel (Figure 7G) and quantified. We noted that MS livers were characterized by increased XBP1 unspliced and spliced mRNA levels. Interestingly, 100 nM SHBG decreased the alternative splicing of XBP1 after 6 h.

### 3.8. SHBG Modulates miRNA Expression in Liver Sections Ex Vivo

**Figure 8:**
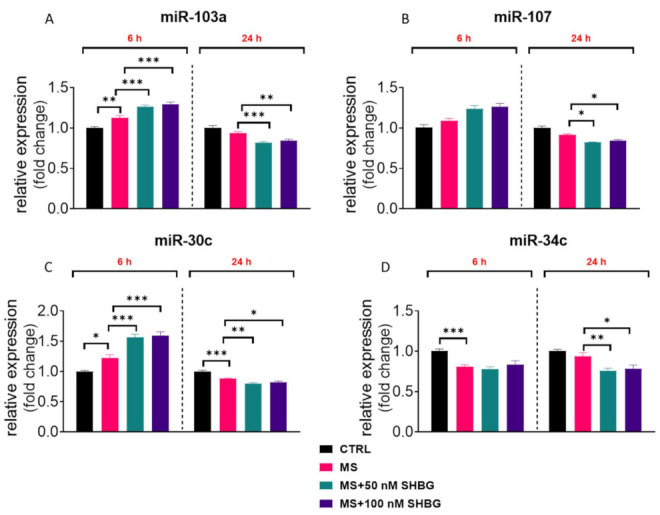
SHBG modulates miRNA expression in liver sections ex vivo. To evaluate the expression of miRNA related to apoptosis and ER stress, liver fragments acquired from tissue samples post mortem were cultured in the presence of 50 and 100 nM SHBG for 6 and 24 h. Next, samples were subjected to RT-qPCR analysis and the expression of miR-103a (**A**), miR-107 (**B**), miR-30c (**C**), and miR-34c (**D**) was established. U6 snRNA was used as a housekeeping gene. The results are presented as mean ± SEM; *n* = 6, * *p* < 0.05, ** *p* < 0.01, *** *p* < 0.001.

In order to evaluate the expression of miRNA related to apoptosis and ER stress, liver fragments acquired from tissue samples post mortem were cultured in the presence of 50 and 100 nM SHBG for 6 and 24 h. Next, samples were subjected to RT-qPCR analysis. We have found that SHBG treatment ameliorated the overexpression of miR-103a (Figure 8A) and miR-107 (Figure 8B) after 24 h of propagation. We also noted an enhanced expression of miR-30c in liver samples treated with SHBG for 6 h, while after 24 h, the expression of that miRNA was reduced (Figure 8C). SHBG treatment for 24 h ameliorated the expression of miR-34c in MS livers (Figure 8D).

## 4. Discussion

The liver plays an essential role in the regulation of carbohydrate, lipid, lipoprotein, and steroid metabolism. The deterioration of hepatic function has been recognized as an initiating factor that might contribute to the development of liver-associated metabolic disorders including metabolic syndrome or type 2 diabetes [31,32]. Alterations in hepatic metabolism lead to the overproduction of glucose and lipids, which in turn lead to oxidative stress, inflammation, IR, and finally ER stress.

Here, we showed that SHBG mitigates palmitate-induced ER stress in hepatocytes as well as the liver of MS individuals. We have found that SHBG in a dose- and time-dependent manner reduces the IRE1α on both the mRNA and protein level. It was showed that under metabolic syndrome or T2D condition, hepatocytes abundantly accumulates unfolded or misfolded proteins in the ER lumen, which leads to the activation of three master regulators of ER stress, including protein IRE1α, PERK, and ATF6 [33]. IRE1a becomes activated during ER stress through the synthesis of x-box transcriptional factor (XBP1), which regulates ER protein folding, trafficking, and maintains ER homeostasis [34]. Total and acetylated sXBP1 protein levels are mostly dependent on XBP1 mRNA splicing, which is regulated by IRE1α [35]. For that reason, we assessed the levels of p-IRE1α as well as sXBP1 and sXBP1 mRNA. Here, we have found that SHBG significantly inhibits the expression of IRE1α and both isoforms of XBP1, i.e., uXBP1 (inactive) isoform and sXBP1 (active), which indicates its protective role against ER stress activation. Moreover, we observed that SHBG downregulates the expression ofCHOP, which triggers ER stress-related apoptosis. It has been documented that under non-stress conditions, the expression of CHOP is reduced, but when ER stress occurs, CHOP is markedly activated and promotes apoptosis [36,37]. The overexpression of CHOP is recognized as an initiating factor for hepatocytes apoptosis related IR in obese and T2D affected mice.

In turn, Selva et al. showed that the overexpression of SHBG in C57BL/ksJ-db/db mice reduces the accumulation of fat by reducing lipogenesis via the dowregualtion of ACC, ACLY, and FASN [38]. Moreover, it was demonstrated that SHBG transgenic mice are protected against NAFLD induced by a high-fat diet. It might be concluded that SHBG exerts a favorable action on liver metabolism by the inhibition of lipogenesis. Herein, we observed a decreased expression of FASN, ACLY, and peroxisome proliferator-activated receptor gamma (PPARG) expression in SHBG-treated hepatocytes. PPARG is a master regulator of adipogenesis and plays a critical role during fatty acids accumulation in the liver of MetS and MS patients or EMS affected horses; thus, its inhibition may prevent lipotoxicicty.

Interestingly, we have found that the differences between 50 nM and100 nM SHBG on cells in many investigated aspects were not statistically significant. It may be due to the fact that SHBG works mainly through binding to its receptor on the cell surface rather than as an intracellular modulator. For that reason, caution should be made in further studies to test dose-dependent effects on other than hepatocytes, as the obtained results can be cell dependent especially while taking into consideration the fact that SHBG is produced by the liver.

We also have found that exogenous SHBG affected the expression of selected miRNAs in both HepG2 cells and liver tissue samples. We observed a significant upregulation of miR-103 and miR-96-5p in HepG2 upon SHBG treatment. Interestingly, it was shown that miR-103 is upregulated in obese individuals; thus, the observed discrepancies requires further research [39]. The overexpression of miR103 was also observed in liver tissue samples after 6 h of treatment. On the other hand, miR-96-5p was shown to enhance proliferation but of cancer cells [40,41,42]. The presented study also revealed that exogenous SHBG decreased the expression of miR-107 in liver fragments from MS individuals. That particular miRNA was shown to be involved in apoptosis progression [43] and tumor progression [44]. In the same samples, we noted an upregulation of miR-30c, which may explain the improved metabolic status of these specimens as decreased levels of that miRNA were observed in cancer patients with shorter overall survival and progression-free survival rates [45].

What is more, our study delivers a new experimental approach in translational studies. Ex vivo studies utilized the unique model of a large animal, horse, which was recently approved by the FDA to study human musculoskeletal disorders and wound healing [1,46,47,48]. This choice is supported by the fact that rodents are a poor model to study SHBG as rodents do not express the SHBG gene in their livers [38,49]. That fact further highlights and supports our thesis that mice are not really an accurate and suitable model for endocrine translational studies to enhance human health and well-being. Their endocrine homeostasis scientifically differs from human, which supports the application of other in vivo models (such as horses) to study SHBG function.

## 5. Conclusions

To summarize, the obtained results confirm that SHBG can be stood as a therapeutic target as its increased levels protect against palmitate-induced lipotoxicicty and ER stress as well as improve the metabolic profile of liver from MS individuals. Thus, SHBG may protect against the development and progression of metabolic syndrome. Our results deliver valuable data, which confirm the hypothesis claiming that SHBG is more that a sex steroid carried and a simple biomarker. Here, we provide evidence that SHBG plays an important role in maintaining hepatocyte homeostasis by the modulation of ER stress and lipotoxicicty through IRE1α.

## Figures and Tables

**Table 1:** Primary antibodies applied in Western blotting.

Protein Abbreviation	Protein Full Name	Manufacturer/Cat No.	Dilution
eIF2α (p-S51)	Eukaryotic translation initiation factor 2 alpha (phospho-S51)	Novus Biologicals/NBP2-67353	1:1000
IRE1α (p-S724)	Inositol-requiring enzyme 1 alpha (phospho-S724)	Biorbyt/orb184380	1:1000
ATF6	Activating transcription factor 6	Novus Biologicals/NBP1-40256	1:1000
SHBG	Sex hormone binding globulin	Biorbyt/orb11366	1:1000
CHOP	DNA damage-inducible transcript 3	Aviva/arp31591	1:1000
Β-actin	Beta-actin	Sigma Aldrich/a5441	1:5000

**Table 2:** Sequence of the primers used for mRNA expression analysis. Hu: human, Eq: equine.

Gene	Full Gene Name	Primer Sequence 5′→3′	Accession No.
*Hu FASN*	Fatty acid synthase	F: CCCAAGCAGGCACACACG R: GGCCTCCGAGGTCTCAG	NM_004104.5
*Hu ACLY*	ATP citrate lyase	F: TGTAACAGAGCCAGGAACCC R: CTGTACCCCAGTGGCTGTTT	NM_001096.3
*Eq ACLY*	ATP citrate lyase	F: CCACTTCAGAGCCCAGACAA R: AACTAGGCCCAGCTTTCCAC	XM_005597396.3
*Hu PPARG*	Peroxisome proliferator-activated receptor gamma	F: AGTCCTCACAGCTGTTTGCCAAGC R: GAGCGGGTGAAGACTCATGTCTGTC	XM_011533844.1
*Hu IRE1A*	Inositol-requiring enzyme 1 alpha	F: CGGCCTCGGGATTTTTGGA R: AGAAAGGCAGGCTCTTCCAC	NM_001433.5
*Hu ATF6*	Activating transcription factor 6	F: ACCTCCTTGTCAGCCCCTAA R: CACTCCCTGAGTTCCTGCTG	NM_007348.4
*Eq ATF6*	Activating transcription factor 6	F: CAGGGTGCACTAGAACAGGG R: AATGTGTCTCCCCTTCTGCG	XM_023640315.1
*Hu HSPA5*	Heat shock protein family A member	F: TGACCAGAATCGCCTGACAC R: TGTCAGCATCTTGGTGGCTT	NM_005347.5
*Hu CHOP*	DNA damage-inducible transcript 3	F: TAAAGATGAGCGGGTGGCAG R: GGATAATGGGGAGTGGCTGG	NM_001195053.1
*Eq CHOP*	DNA damage-inducible transcript 3	F: AGCCAAAATCAGAGCCGGAA R: GGGGTCAAGAGTGGTGAAGG	XM_001488999.4
*Hu XBP1*	X-box binding protein 1	F: CGCGGATCCGAATGAAGTGAGGCCAGTG R: GGGGCTTGGTATATATGTGG	XM_014742035.2
*Eq XBP1*	X-box binding protein 1	F: TTACGCGAGAAAACTCATGGCC R: GGGTCCAAGTTGAACAGAATGC	XM_014742035.2
*Hu SHBG*	Sex hormone binding globulin	F: GCTGATTATGGAGAGCAGAGG R: GGTCATGACAGCGATAGGCT	NM_001146281.3
*Eq SREBP1C*	Sterol regulatory element-binding transcription factor 1	F: TCAGCGAGGCGGCTTTGGAGCAG R: CATGTCTTCGATGTCGGTCAG	XM_008542859.1
*Eq BIP*	binding immunoglobulin protein	F: CTGTAGCGTATGGTGCTGCT R: CATGACACCTCCCACGGTTT	XM_023628864.1
*Hu GAPDH*	Glyceraldehyde 3-phosphate dehydrogenase	F: GTCAGTGGTGGACCTGACCT R: CACCACCCTGTTGCTGTAGC	NM_001289746.1
*Eq GAPDH*	Glyceraldehyde 3-phosphate dehydrogenase	F: GATGCCCCAATGTTTGTGA R: GATGCCCCAATGTTTGTGA	NM 001163856.1

**Table 3:** Sequence of the primers used for miRNA expression analysis.

miRNAs	Primer Sequence 5′→3′
miR-103	AGCAGCATTGTACAGGGCTATGA
miR-93-5p	CAAAGTGCTGTTCGTGCAGGTAG
miR-34a	TGGCAGTGTCTTAGCTGGTTGT
miR-30c	TGTAAACATCCTACACTCTCAGC
miR-107	AGCAGCATTGTACAGGGCTATCA
miR-34a	AGGCAGTGTAGTTAGCTGATTGC

## Data Availability

The datasets generated during and/or analysed during the current study are available from the corresponding author on reasonable request.
